# Aortic Stenosis Prognostication in Patients With Type 2 Diabetes: Protocol for Testing and Validation of a Biomarker-Derived Scoring System

**DOI:** 10.2196/13186

**Published:** 2019-08-12

**Authors:** Suresh Giritharan, Felino Cagampang, Christopher Torrens, Kareem Salhiyyah, Simon Duggan, Sunil Ohri

**Affiliations:** 1 Institute of Developmental Sciences Faculty of Medicine University of Southampton Southampton United Kingdom; 2 Wessex Cardiac Centre University Hospitals Southampton Southampton United Kingdom

**Keywords:** aortic stenosis, myocardial fibrosis, type 2 diabetes mellitus, biomarkers, ventricular remodelling, aortic valve replacement

## Abstract

**Background:**

Type 2 diabetes mellitus (T2DM) has been established as an important independent risk factor for aortic stenosis. T2DM patients present with a higher degree of valve calcification and left ventricular dysfunction compared to patients without diabetes. This may be due to an increase in incidence and severity of myocardial fibrosis. Currently, there is no reliable method of determining the optimal timing of intervention for a patient with asymptomatic aortic stenosis or predicting when a patient will become symptomatic. Research into serum biomarkers to predict subclinical onset and track progression of aortic stenosis is hampered by the multimodal nature of the pathological processes ultimately responsible for aortic stenosis.

**Objective:**

The aim of this study is to prove that an approach using a combination of serum biomarkers and the echocardiographic parameter global longitudinal strain (GLS) can be used to establish baseline status of fibrocalcific aortic valve disease, predict rate of progression, and quantitatively assess any regression of these processes following aortic valve replacement in patients with T2DM.

**Methods:**

Validated serum biomarkers for the separate processes of calcification, inflammation, oxidative stress and fibrosis can be used to quantify onset and rate of progression of aortic stenosis. This, in combination with the echocardiographic parameter GLS, can be compared with other objective investigations of calcification and fibrosis with the aim of developing a quick, noninvasive one-stop assessment of aortic stenosis in patients with T2DM. The serum biomarkers BNP (B-type natriuretic peptide), Gal-3 (Galectin-3), GDF-15 (growth differentiation factor-15), sST2 (soluble suppression of tumorigenicity 2), OPG (osteoprotegerin), and microRNA 19b and 21 will be sampled from patients undergoing aortic valve replacement (with and without T2DM), patients with T2DM but without aortic valve disease and healthy volunteers. These patients will also undergo computed tomography (CT) scans for calcium scoring, magnetic resonance imaging (MRI) to quantify myocardial fibrosis, and myocardial strain imaging with speckle-tracking echocardiography. Samples of calcified native aortic valve and a biopsy of ventricular myocardium will be examined histologically to determine the quantity and distribution of calcification and fibrosis, and the secretome of these tissue samples will also be analyzed for levels of the same biomarkers as in the serum samples. All patients will be followed up with in 3 months and 12 months for repeat blood sampling, echocardiography, and CT and MRI imaging to assess disease progression or regression. The results of tissue analysis and CT and MRI scanning will be used to validate the findings of the serum biomarkers and echocardiographic assessment.

**Results:**

Using all of the information gathered throughout the study will yield a ranking scale for use in the clinic, which will provide each patient with a fibrocalcific profile. This can then be used to recommend an optimal time for intervention.

**Conclusion:**

A reliable, validated set of serum biomarkers combined with an inexpensive bedside echocardiographic examination can now form the basis of a one-stop outpatient-based assessment service, which will provide an accurate risk assessment in patients with aortic stenosis at first contact.

**International Registered Report Identifier (IRRID):**

PRR1-10.2196/13186

## Introduction

Aortic stenosis (AS) remains the most common valvular disease in adults, with a prevalence of 5% in people over the age of 65 years [1]. While type 2 diabetes mellitus (T2DM) has been established as an independent risk factor for this disease, little is understood about the precise nature of this association. Initially, it was attributed to the same inflammatory processes involved in the development of coronary artery disease (CAD) [2]. This theory is now in doubt as modulation of coronary risk factors do not reduce the incidence of aortic stenosis in these patients [3]. Advanced stages of AS warrants surgical aortic valve replacement (AVR) [4]. Despite the aid of echocardiographic parameters allowing for delineation of mild, moderate and severe aortic stenosis, the single prevailing indication for surgical intervention is the severity of symptoms [5].

The physiological narrowing of the valve which limits the outflow of blood is the end result of a complex array of cellular mechanisms that culminate in two distinct but intertwined pathological processes: calcification and fibrosis [6]. These mechanisms are also responsible for the stiffening and loss of elasticity of ventricular muscle due to abnormal deposition of collagen, a process termed myocardial fibrosis [7]. Currently, there is no reliable method of ascertaining if, or how soon, a patient with asymptomatic AS will become symptomatic. Given that these cellular modulations occur insidiously_presumably over a duration of time prior to the onset of symptoms_it is likely that the ventricular myocardium will have sustained a degree of fibrotic insult prior to identification of the need for aortic valve replacement. This current approach to guiding therapy has the potential pitfall of depriving these patients of the therapeutic effect of early reverse remodeling of the ventricle. This is a process where stiffened, fibrotic myocardium has the potential to regress to its initial elastic, nonhypertrophic state once ventricular afterload (secondary to stiffening by calcification and fibrosis) is reduced by AVR [8].

As the presence of T2DM has been shown to accelerate the progression of aortic stenosis, it is not unreasonable to conclude that patients with T2DM who develop severe aortic stenosis warranting AVR may have more advanced underlying myocardial fibrosis. This may not be amenable to the beneficial phenomenon of reverse-remodeling following replacement of the valve; such T2DM patients may benefit from early AVR. A reliable strategy of tracking the cadence of disease progression and susceptibility to reverse-remodeling is therefore necessary, as this would optimize timing of surgery for high-risk patients. We hypothesize that a method utilizing biomarkers for the tracking of underlying cellular processes culminating in aortic stenosis shows promise in achieving this.

## Methods

### Presentation of the Hypothesis

The identification of a specific serum biomarker which can provide both an insight to the static state of a pathological process and track its progression (and subsequent regression following appropriate intervention) has proved challenging. This is due to the multimodal nature of the mechanisms which ultimately culminate in stiffening of the aortic valve. However, as a result of various studies of calcification and fibrosis in the heart, there is consensus that four core subprocesses are synergistically responsible in achieving the endpoint of aortic stenosis: valve mineralization (calcification), local inflammation, oxidative stress and ventricular fibrosis [9]. Within the domain of each subprocess, several promising biomarkers have shown acceptable specificity [10]. Here, we hypothesize that an approach using a combination of serum biomarkers and the echocardiographic parameter global longitudinal strain (GLS), a functional marker which will be discussed later, can be used to establish baseline status of fibrocalcific aortic valve disease, predict rate of progression and quantitatively assess any regression of these processes following AVR in patients with T2DM.

### Serum Biomarkers

**Figure 1:**
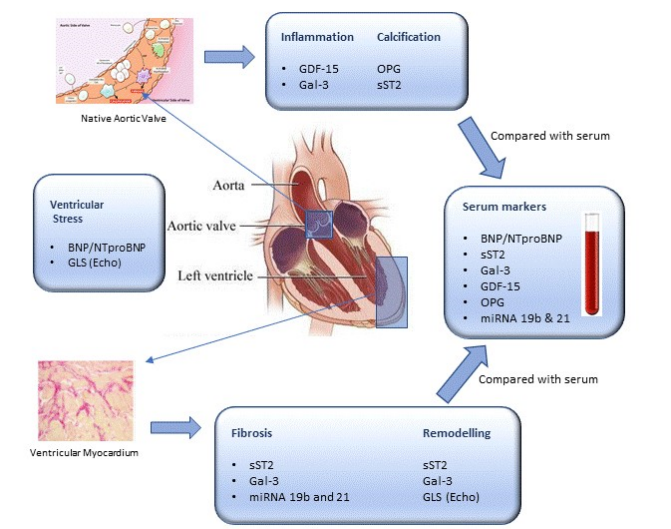
Location of biomarkers of inflammation, calcification, fibrosis, ventricular stress and remodelling in aortic valve and myocardial tissue which will be compared to levels in serum. BNP, Brain Natriuretic Peptide; Gal-3, Galectin-3; GDF-15, Growth Differentiation Factor 15; GLE, Global Longitudinal Strain; miRNA, micro ribonucleic acid; OPG, Osteoprotegerin; sST2, Soluble ST2.

Figure 1 provides an overview of the site of expression of relevant biomarkers. BNP (B-type natriuretic peptide), and its prohormone NT-proBNP (n-terminal pro brain natriuretic peptide), are neurohormones and markers of stretching of the heart muscle. BNP is secreted by cardiomyocytes following volume and pressure changes in the left ventricle. NT-proBNP in particular demonstrates an incremental relationship with the severity of aortic stenosis. Although in isolation this biomarker is unable to distinguish between the pathologies of calcification and fibrosis, it is valuable in ascertaining the cumulative consequence of ventricular dysfunction and has predictive value [11].

sST2 (soluble suppression of tumorigenicity 2) is a member of the IL1 (interleukin-1) receptor family and is a marker of ventricular remodeling. sST2 levels increase with myocardial stress and have been demonstrated to correlate quantitatively with myocardial ischemia and heart failure. It is of particular use as it correlates with echocardiographic assessment of diastolic dysfunction and has been demonstrated to correlate with heralding the onset of new symptoms in previously asymptomatic aortic stenosis patients. High levels of sST2 are an independent predictor of mortality following aortic valve replacement surgery [12].

Gal-3 (Galectin-3) is a beta-galactoside-binding lectin and a marker of inflammation and fibrosis of the myocardium, but it is also responsible for other processes such as regulating T-cell function and apoptosis. Gal-3 has a limited role in predicting progression of ventricular stiffening as it is also upregulated in other conditions such as renal dysfunction. However, when considered in tandem with BNP, it has shown to have significant predictive properties in the progression of heart failure [13].

The microRNAs 21 and 19b are specific small RNAs that are 19-25 nucleotides long which are postulated to regulate gene expression related to various cellular mechanisms underpinning cardiovascular function. Although assessment of these micro-RNAs is still in early stages, microRNA-21 has shown good correlation with myocardial fibrosis. MicroRNA-19b has shown promise in determining increased myocardial collagen cross-linking in patients with aortic stenosis [14].

GDF-15 (growth differentiation factor-15) is a stress-responsive cytokine that is sensitive to inflammatory changes and apoptosis. It is abundant in cardiomyocytes, adipocytes and macrophages and has demonstrated good predictive value in heart failure, vascular calcification and endothelial dysfunction. Like Gal-3, it is upregulated in renal dysfunction and additionally in obesity and insulin-resistant states such as diabetes. Although recent work has established GDF-15 as a useful prognostic and diagnostic marker of cardiometabolic disease in patients with diabetes, reference ranges correlating to severity have not yet been established [15].

OPG (osteoprotegerin) is a member of the tumor necrosis factor receptor family and is expressed in early arteriosclerotic lesions and vascular smooth muscle cells. It has an important role in neocalcification, acting as a decoy receptor by binding to the RANKL (receptor activator kappa-B ligand) nuclear factor and inhibiting the receptor activity of RANK (receptor-activated kappa-B). This thus inhibits osteoclastic activity, which ultimately leads to calcification. Modulation of this pathway by the action of anti-Ghrelin antibodies suppressing OPG expression is of particular interest in seceding calcification [16].

### Global Longitudinal Strain as a Complimentary Functional Marker

Speckle-tracing echocardiography is a relatively novel echocardiographic technique which has been shown to be more sensitive than conventional assessments of ejection fraction (EF) in the assessment of minute, regional wall motion abnormalities (RWMA) and left ventricular function. It correlates well with the presence and magnitude of myocardial fibrosis when compared alongside cardiac magnetic resonance, as assessed through T1-mapping and late gadolinium enhancement as quantification techniques. This noninvasive imaging modality can serve as an additional functional marker alongside the serum biomarkers mentioned above in the assessment of severity of aortic calcification and in the evaluation of reverse-remodeling postoperatively [17].

### Testing the Hypothesis

**Figure 2:**
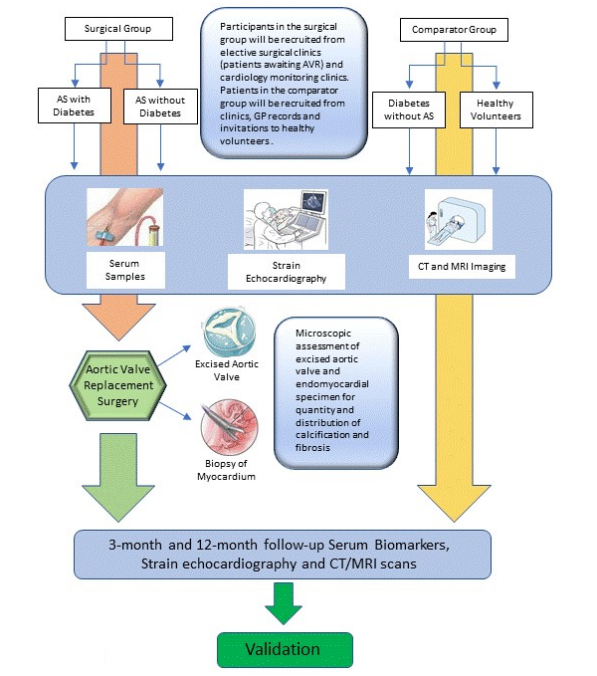
Testing the hypothesis. Preoperative serum biomarkers and echocardiography will be compared to CT and MRI imaging to quantify calcification and fibrosis. Samples of valve and myocardial tissue will be examined for calcification and fibrosis and compared to serum biomarkers. Follow-up investigations will assess if upregulation or downregulation of biomarkers show reliable clinical correlation with disease progression or regression. AS: aortic stenosis; AVR: aortic valve replacement; CT: computed tomography; MRI: magnetic resonance imaging.

Figure 2 outlines the process of biomarker testing in the relevant patient cohorts. Gold standard assessments of aortic valve calcification and myocardial fibrosis are direct microscopic inspection of the excised aortic valve, following AVR, and a biopsy specimen of the ventricular myocardium, respectively [18]. Noninvasive imaging techniques done preoperatively, Computed Tomographic (CT) Calcium Scoring for aortic valve calcification and Late Gadolinium Enhancement magnetic resonance imaging (MRI) for myocardial fibrosis, will corroborate the findings of direct inspection. These results will be used to calibrate serum biomarkers and echocardiographic findings for the subset of patients with severe AS undergoing surgery.

Healthy volunteers and patients with milder, asymptomatic forms of the disease will be recruited from outpatient cardiology surveillance clinics to undergo serum biomarker sampling and strain echocardiograms. They will then undergo CT and MRI scans to investigate the relevance of these markers against objective imaging findings. This will enable the establishment of baseline levels in healthy volunteers and grading of the markers in mild, moderate and severe forms of disease.

Finally, postoperative patients will be followed up with in 3 months and 12 months following surgery where they will once again undergo serum biomarker sampling and strain echocardiography to assess for evidence of reverse-remodeling. Again, CT scans for calcium scoring and MRI scans will be repeated to validate the significance of any downregulation of these biomarkers.

The combination of biomarker and echocardiographic data encompassing the entire spectrum of the disease from healthy volunteers to patients with severe disease as well as disease monitoring in postoperative patients will yield a ranking scale for use in the clinic. This scale, once validated for reproducibility in an appropriately powered study, will provide each patient with a fibrocalcific profile which can then be used to recommend an optimal time for intervention.

## Results

Enrollment has not commenced for this study. Ethical approval was obtained from South Central - Hampshire A Research Ethics Committee (United Kingdom – REC: 18/SC/1062, IRAS: 024397). Funding was obtained from the Wessex Heart Surgery Charitable Fund (registered charity #1051543).

## Discussion

A reliable, validated set of serum biomarkers combined with an inexpensive bedside echocardiographic examination can now form the basis of a one-stop outpatient-based assessment service in providing an accurate risk assessment in patients with AS at first contact. In patients with diabetes, the role of such a service can be expanded to an essential screening service given the less favorable prognosis of aortic stenosis in this demographic.

## Abbreviations

AS: aortic stenosis
AVR: aortic valve replacement
BNP: brain natriuretic peptide
CAD: coronary artery disease
CT: computed tomography
EF: ejection fraction
Gal-3: Galectin 3
GDF-15: growth differentiation factor 15
GLS: global longitudinal strain
IL1: Interleukin-1
MRI: magnetic resonance imaging
NT-proBNP: n-terminal pro brain natriuretic peptide
OPG: osteoprotegerin
RANKL: receptor-activated kappa B ligand
RNA: ribonucleic acid
RWMA: regional wall motion abnormality
sST2: soluble suppression of tumorigenicity 2
T2DM: type 2 diabetes mellitus
